# *Notch4* is essential for the maintenance of vascular homeostasis in the young adult pituitary posterior lobes

**DOI:** 10.1038/s41598-025-17225-5

**Published:** 2025-08-24

**Authors:** Noriyoshi Takebe, Yoshito Sugita, Shigeki Takada, Kazue Muraki, Sally L. Dunwoodie, Masato Hojo, Susumu Miyamoto, Yoshiki Arakawa, Kenji Tanigaki

**Affiliations:** 1 Department of Neurosurgery, Kyoto University Graduate School of Medicine, Kyoto, Japan; 2 Research Institute, Shiga Medical Center, Shiga, Japan; 3 Clinical Research Center, Shiga General Hospital, Shiga, Japan; 4 Department of Neurosurgery, Shiga General Hospital, Shiga, Japan; 5 Victor Chang Cardiac Research Institute, Sydney, Australia; 6 School of Clinical Medicine, Faculty of Medicine and Health, University of New South Wales, Sydney, Australia

**Keywords:** Notch, Pituitary, Vessels, Development, VEGF, RBP-J, Angiogenesis, Differentiation

## Abstract

Notch signaling is critical for vascular development. However, the functions vary significantly depending on organs and the developmental stage. The adult vasculature is less susceptible to Notch signaling inhibition. Vessels in the adult pituitary posterior lobes maintain characteristics of immature vessels, such as sensitivity to VEGF. Here, we show that *Notch4* deficiency caused reduction of vascular density, vascular branch length and the number of branching points and the larger radius vessels in the adult pituitary posterior lobes using the automated vascular image analysis method. Upregulation of NR2F2 by *Notch4* loss induced arteriovenous identity deficiency. *Notch4* has been reported to have dual roles on the regulation of RBP-J-mediated transcription. DAPT is a γ-secretase inhibitor, which inhibits Notch signaling. DAPT-treated adult pituitary posterior lobes showed similar vascular phenotypes to that of *Notch4* knockout mice except for the length of affected larger radius vessels, suggesting the importance of RBP-J-mediated transcription in vascular maintenance of the adult pituitary posterior lobes. These data indicate that the maintenance of adult pituitary posterior lobe vasculature requires Notch signaling.

**Supplementary Information:**

The online version contains supplementary material available at 10.1038/s41598-025-17225-5.

## Introduction

Notch and vascular endothelial growth factor (VEGF) signaling play pivotal roles in various aspects of vascular development. Vascular expansion occurs via angiogenesis which is the process by which new blood vessels form from existing vessels. Retinal vasculature studies have shown that the feedback loop of VEGF and Notch signaling control angiogenesis through the regulation of tip cell formation^1–6^. VEGF-A/VEGF receptor 2 (VEGFR2) signaling promotes endothelial cell sprouting, proliferation and tip cell induction in the early phase of vascular development^7,8^. VEGF-A/VEGFR2 signaling induces the expression of a Notch ligand, delta-like 4 (Dll4), which activates Notch1 receptor of surrounding endothelial cells and suppresses their tip cell behavior. Expanded vasculatures are remodeled by pruning, stabilized and differentiated into arteries and veins. VEGF/Notch signaling are required for the arterial differentiation^9–11^. In the growth phase, VEGF is indispensable for the survival of blood vessels. VEGF inhibition induces vessel regression^12,13^and Notch inhibition prevents vessel pruning^14^. In contrast, most of adult blood vessels are generally stable, and do not require VEGF for their survival^15,16^. In the adult mature vessels, Notch activation suppresses the expression of VEGFR2 and activation of mitogen-activated protein kinase (MAPK)/ extracellular signal regulated kinase (ERK) signaling activation, leading to the stability and the VEGF independence^17–20^. This role of Notch signaling in maintaining endothelial cell quiescent is restricted to veins and perivenous capillaries^18^. However, adult arterial fate is not affected by loss of Notch signaling, although Notch signaling is required for the differentiation of artery in embryonic stages^9,11,18^. Thus, the functions of VEGF and Notch signaling differ between developing and mature blood vessels. Vessels in the adult pituitary have been reported to be susceptible for the loss of VEGF^21,22^ suggesting the existence of pituitary-specific vascular heterogeneity.

Notch4 is specifically expressed endothelial cells^23,24^ and Notch4 and Notch1 cooperatively regulate vessel development^25–27^. Notch1 and Notch4 activation upregulates RBP-J-mediated transcription^28^. However, it has been reported that the extracellular domain and intracellular domain of Notch4 have opposing functions on RBP-J mediated transactivation^26^. To elucidate the roles of Notch4 in the adult pituitary vasculature, we examined the pituitary posterior lobe vessels of *Notch4* knockout (KO) mice and compared their phenotypic characteristics with those of DAPT-treated mice, given its ability to inhibit Notch signaling in vivo.

## Results

### Changes in blood vessels of the adult pituitary posterior and intermediate lobes in the absence of *Notch4*

**Fig. 1:**
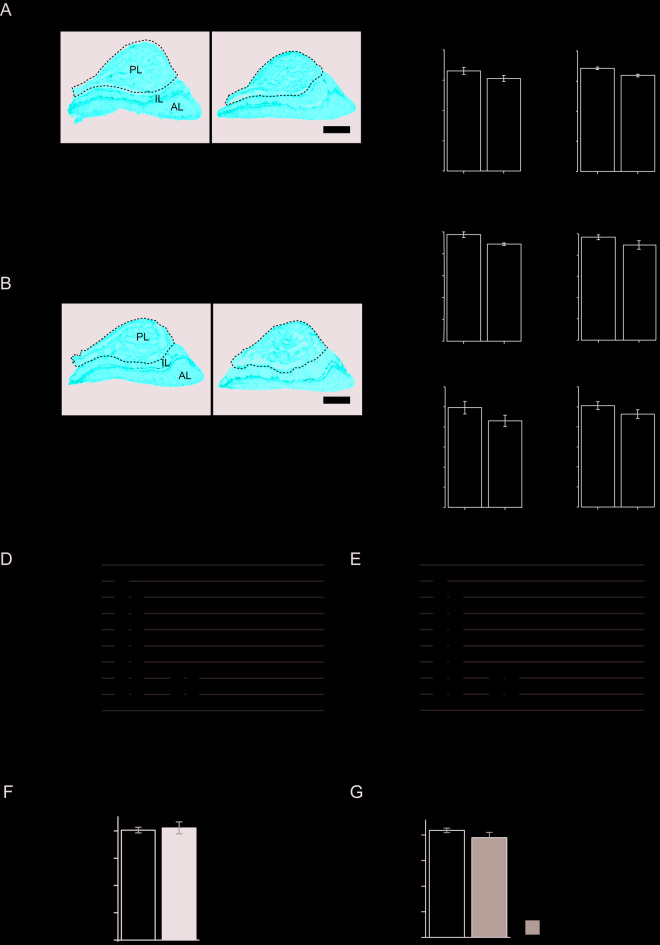
Aberrant vascular morphogenesis of the pituitary posterior lobes of Notch4-deficient mice or DAPT-treated mice. **(A**, ** B)** Immunofluorescent analysis for isolectin B4 of the pituitary posterior lobes of control mice, Notch4 ^−/−^ mice (A), and three-day DAPT-treated mice(B). **(C**, ** D**, **E**, **F)** Binary images of A-B, E-F. Quantification of vascular density, densities of branch numbe, branch points (C), the branch length distributions (D, E) and the volumes of the pituitary posterior lobes (F, G). Scale bars = 300 μm (A, B). Data are represented as means ± S.E.M. *P = 0.0066, **P = 0.0054, ***P = 0.010, ****P = 0.0068, ^#^*P* = 0.0076, ^##^*P* = 0.013 (Student’s t test), ^###^*P* = 0.0029 (Bonferroni correction).

**Fig. 2:**
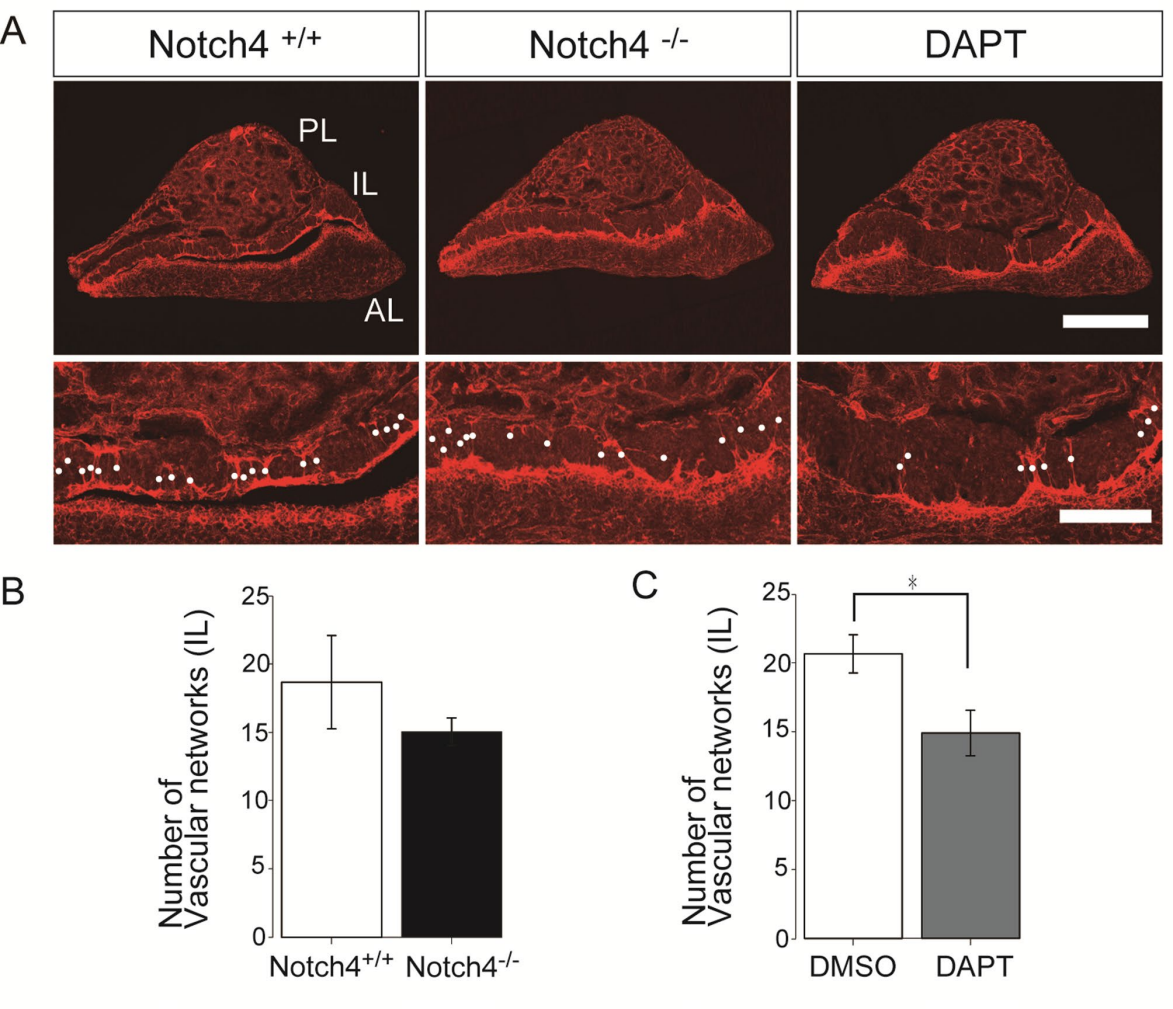
Aberrant vascular morphogenesis of the pituitary intermediate lobes of Notch4-deficient mice or DAPT-treated mice. **(A)** Representative sagittal sectional images of pituitaries of control mice, Notch4 ^−/−^ mice, and three-day DAPT-treated mice. Immunofluorescent analysis for isolectin B4. Intermediate lobe regions in the upper panels are shown at higher magnification in the lower panels. White dots indicate capillary plexus. **(B**, **C)** Quantification of density of vascular networks. Scale bars = 400 μm (upper panels), 200 μm (lower panels). Data are represented as means ± S.E.M. *P = 0.041(Student’s t test).

**Fig. 3:**
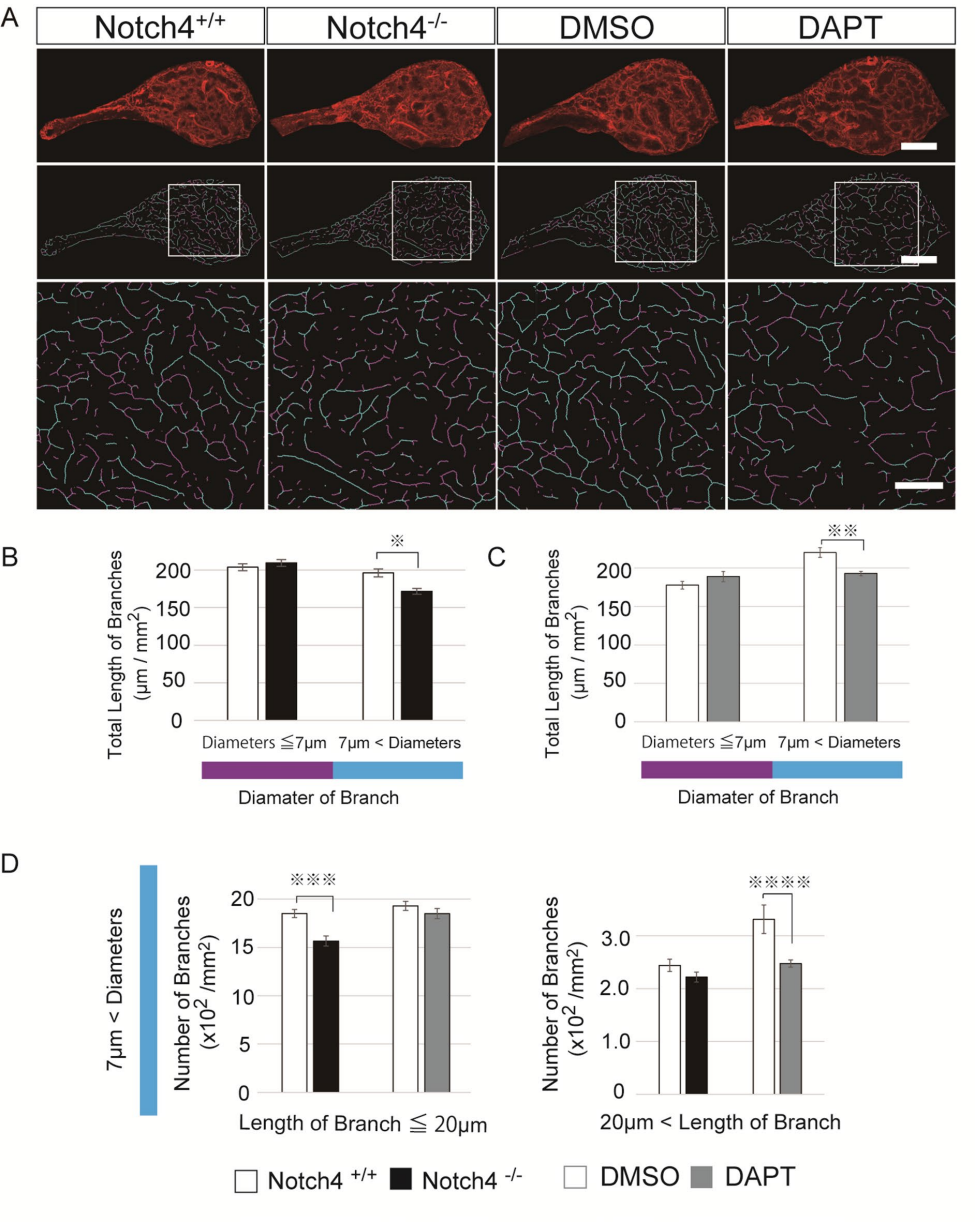
Aberrant vascular distribution in the pituitary posterior lobes of Notch4-deficient mice or DAPT-treated mice. **(A)** Immunofluorescent analysis for isolectin B4 of the pituitary posterior lobes of control mice, Notch4 ^−/−^ mice, and three-day DAPT-treated mice (upper panels). Vessel radius map of the pituitary posterior lobes (Intermediate panels). Magenta and blue represent smaller radius vessels (≤ 7 μm) and larger radius vessels (> 7 μm). Magnified views of the boxed regions from the intermediate panels are provided in the lower panels. **(B-D)** Quantification of total length of smaller radius vessels (≤ 7 μm) (magenta) and larger radius vessels (> 7 μm ) (blue) (B and C) and shorter length branches (≤ 20 μm) with larger radius vessels (> 7 μm ) and longer length branches (> 20 μm) with larger radius vessels (> 7 μm) (D). Scale bars = 200 μm (upper and intermediate panels), 100 μm (lower panels). Data are represented as means ± S.E.M. *P = 0.0058, **P = 0.0047, ***P = 0.0031, ****P = 0.017 (Student’s t test).

To evaluate the effects of *Notch4* deficiency on vascular networks of the pituitary, we established automative quantitative imaging analysis workflow for vascular parameters: branch density, branch length, the number of branch points and branch radii^29,30^ (Supplementary Fig. 1). First, we applied a segmentation approach to the images of Isolectin B4-positive vascular endothelial cells by binarization to quantify vascular area (Fig. 1A). Vascular density was calculated by dividing the quantified vascular area by the total area of the pituitary posterior lobe. Next, centerline pixels of segmented vasculature were extracted by skeletonization to quantitate branch length and the number of branch points. The radius of the blood vessel was defined as the distance from the vascular centerline pixel to the nearest point on the vessel wall. The quantification showed that loss of *Notch4* leads to decrease in vascular density, vascular branch length and the number of branching points in the adult pituitary posterior and intermediate lobes, while having no effects on the size of posterior lobe (Figs. 1A, C and F and 2A and B). Next, we analyzed the distribution of branch length. A repeated measures ANOVA revealed a significant overall difference between *Notch4* KO and control mice in vessel number distribution across the vessel length (genotype-by-branch length interaction, F_3,24_ = 9.06, *n* = 5 per group, *P* = 0.0003 (repeated two-way ANOVA) (Fig. 1D). Specific reduction in short branches was observed in the absence of *Notch4*, although subsequent post-hoc analysis with Bonferroni correction did not show significant differences. We next examined the vascular radii in *Notch4* KO mice. Loss of *Notch4* results in specific decrease in larger radius vessels (> 7 μm) compared with smaller radius vessels (≤ 7 μm) (Fig. 3A and B). This reduction in larger blood vessels was specifically observed only in vessels shorter than 20 μm (Fig. 3D).

### Changes in blood vessels of the DAPT-treated pituitary pituitary posterior and intermediate lobes

*Notch4* has been reported to have dual roles on *RBP-J*-mediated transcription^26,28^. To examine the effects of the inhibition of *RBP-J*-mediated transcription, we administrated DAPT 100 mg/kg s.c. twice a day for 3 days. DAPT treatment reduced vascular density, vascular branch length and the number of branching points in the adult pituitary posterior and intermediate lobes but did not affect the size of posterior lobe (Figs. 1B, C and G and 2A and C) and specific reduction in short branches was also observed in DAPT-treated-pituitary posterior lobes (treatment-by-branch length interaction, F_3,24_ = 16.11, *n* = 5 per group, *P* < 0.0001 (repeated two-way ANOVA) (Fig. 1E). DAPT treatment also leads to specific decrease in larger radius vessels (> 7 μm) (Fig. 3A and C), but this reduction was observed only in vessels longer than 20 μm (Fig. 3D). These data show that the pituitary vascular phenotype of *Notch4* KO mice is very similar to that induced by DAPT-mediated Notch inhibition. However, the phenotypes were not completely consistent, in terms of the length of the affected larger radius blood vessels.

### The effects of *Notch4* deficiency or DAPT treatment on arterial identity in the pituitary posterior lobes

**Fig. 4:**
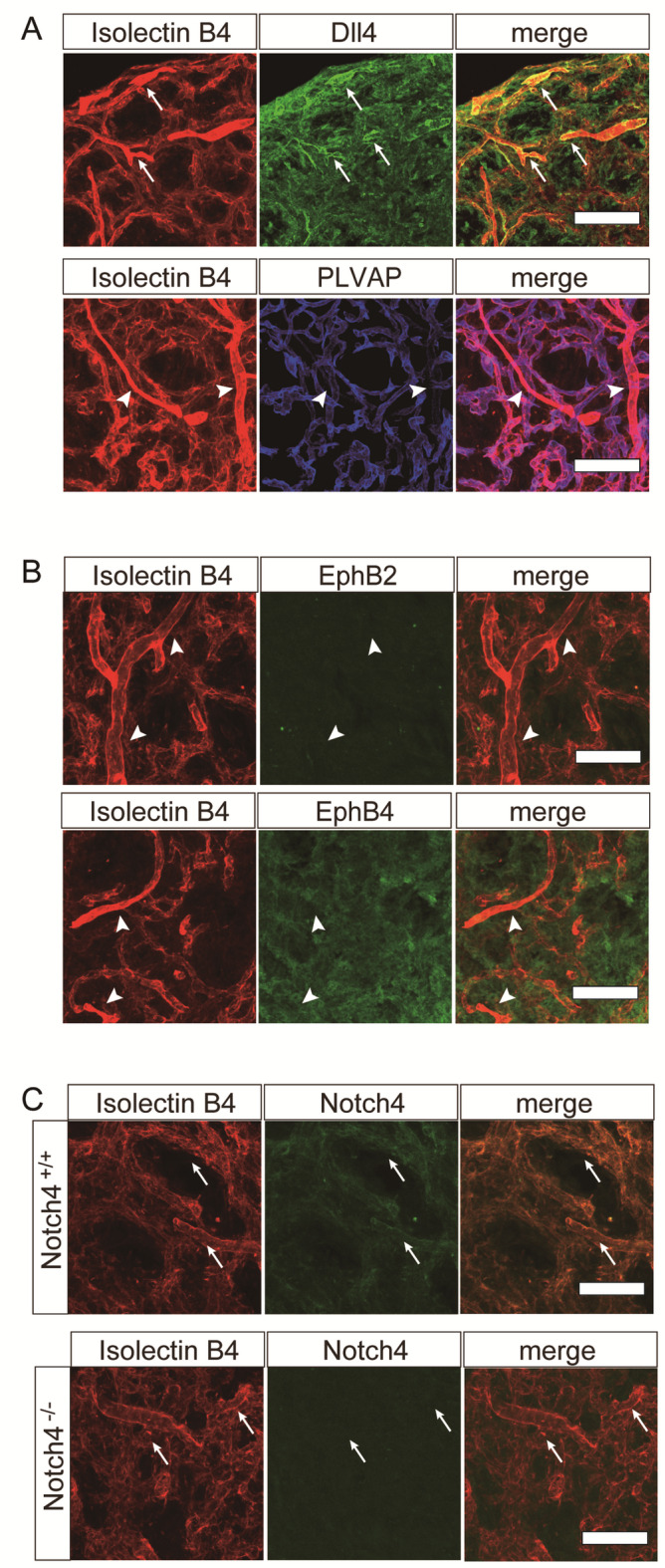
Immunofluorescent analysis of vascular networks in the pituitary posterior lobe. **(A-C)** Immunofluorescent analysis for Isolectin B4 (red) (A, B and C), Dll4 (green) (A), EphB2 (green) (B), EphB4 (green) (B), Notch4 (green) (C) and PLVAP (blue) (A). Larger radius vessels strongly labeled with Isolectin B4 are Dll4-positive (arrows) and PLVAP-negative (arrow heads) (A), EphB2-negative (arrow heads) and EphB4-negative (arrow heads) (B). (C) shows that Notch4 expressed in Isolectin B4-positive vessels in the pituitary posterior lobe but not in Notch4 KO mice. Scale bars = 50 μm (A, B and C).

**Fig. 5:**
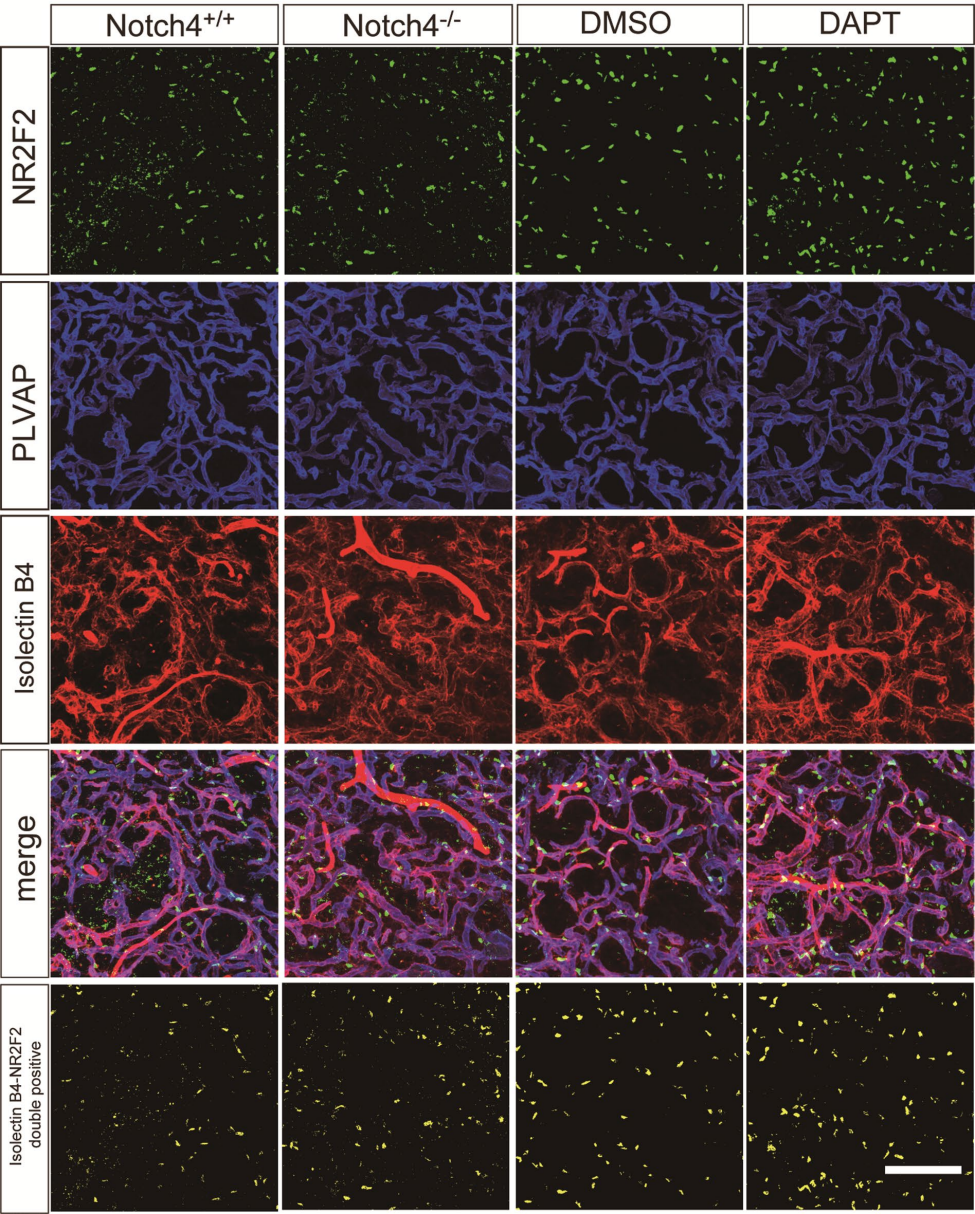
Expression of NR2F2, a venous endothelial marker, in the pituitary posterior lobes of Notch4-deficient mice or DAPT-treated mice. Immunofluorescent analysis for NR2F2 (green), PLVAP (blue), Isolectin B4 (red) of the pituitary posterior lobes of control mice, Notch4 ^−/−^ mice and three-day DAPT-treated mice. NR2F2 + and Isolectin B4 + double-positive cells are represented in pseudocolored yellow in the lowest panels. Scale bars = 100 μm.

Specific reduction of larger radius vessels (> 7 μm) was observed in the posterior lobes of Notch4 KO and DAPT-treated pituitaries. To characterize these vessels, we performed immunofluorescent studies, which revealed that IslectinB4-strongly positive larger radius vessels are Dll4, an arterial endothelial marker-positive and PLVAP-negative (Fig. 4A). PLVAP has been reported to be specifically expressed in fenestrated capillaries in the pituitary posterior lobes^31^. These data show larger radius vessels contain arteries. Neither Ephrin-B2 (EphB2), an arterial marker nor EphB4, a venous marker is expressed in IslectinB4-positive vessels, indicating that these markers are not suitable for distinguishing arteries from veins in the pituitary (Fig. 4B) The loss of Notch4 expression in endothelial cells suggests a cell-autonomous mechanism (Fig. 4C). Next, we performed immunohistochemical analysis of PLVAP and NR2F2(COUP-TF2), a venous marker. Neither loss of *Notch4* nor DAPT treatment affected PLVAP-positive fenestrated capillaries (Fig. 5). As NR2F2 is expressed in both endothelial and non-endothelial cells, we performed co-localization analysis with the endothelial marker Isolectin B4 to specifically investigate NR2F2 expression within the vasculature. The loss of *Notch4* or DAPT treatment increased the number of Isolectin B4^+^, NR2F2^+^ double positive cells (Fig. 5), which suggests that *Notch4* deficiency or DAPT treatment leads to upregulation of NR2F2, a venous marker and arteriovenous identity deficiency. Taken together, loss of arteriovenous identity might cause the reduction in larger radius vessels in *Notch4* KO mice or DAPT-treated mice.

### The effects of *Notch4* deficiency or DAPT treatment on the Notch-related genes in the pituitary posterior lobes

**Fig. 6:**
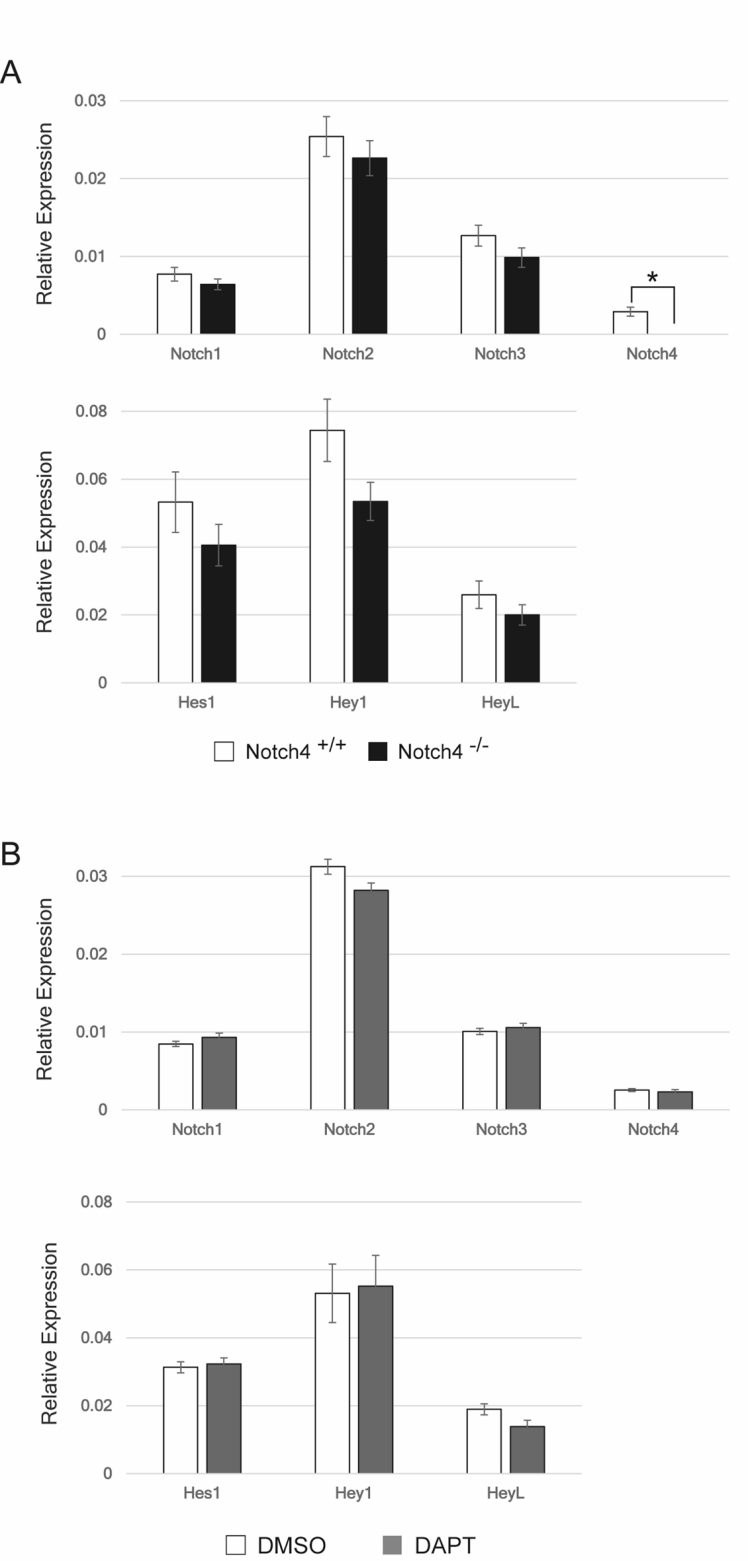
Quantitative real-time RT-PCR analysis of Notch-related genes in the pituitary posterior lobe. **(A and B)** Quantitative real-time RT-PCR of Notch1, Notch2, Notch3, Notch4, Hes1, Hey1 and HeyL in the adult pituitary posterior lobe of Notch4 KO mice and DAPT-treated mice. Values have been normalized to GAPDH abundance. Data are represented as means ± S.E.M. *P = 0.0064 (D).

Quantitative real-timePCR analysis shows significant reduction of Notch4 expression in *Notch4* KO mice (Fig. 6A). However, neither loss of Notch4 nor DAPT treatment affected the expression of Notch1-4, Hes1, Hey1 and HeyL, which are well-established Notch target genes (Fig,6A and B). These data suggest we could not exclude the possibility that the effects of Notch4 deficiency or DAPT treatment on the vasculature of the pituitary posterior lobe may occur through mechanisms independent of canonical Notch signaling pathways. To further investigate this hypothesis, future studies employing endothelial-specific *RBP-J* conditional KO mice or endothelial-specific *dominant-negative MAML1* transgenic mice will be necessary.

## Discussion

Like VEGF signaling, Notch signaling is known to regulate differentially immature versus mature postnatal vessels. Mature adult vascular endothelial cells are less susceptible to Notch signaling inhibition^15,16,18,32^. However, here, we show that *Notch4* deficiency or DAPT treatment caused reduction of vascular density, vascular branch length and the number of branching points and the larger radius vessels in the adult pituitary posterior lobes using the automated vascular image analysis method. The expression of NR2F2 was increased by Notch4 deficiency or DAPT treatment, suggesting arteriovenous identity deficiency. These data suggest that adult vasculature of the pituitary posterior lobes remains sensitive to Notch signaling inhibition, which impairs vascular network maintenance. However, we cannot exclude the possibility of Notch4 involvement during development, nor can we rule out that the observed phenotypes are secondary consequences of its deficiency in non-endothelial cells. To further validate this conclusion and delineate the endothelial-specific roles of Notch4 in vascular maintenance, future studies utilizing endothelial-specific *Notch4* conditional KO mice will be necessary.

*Notch4* has been reported to have dual roles on the regulation of RBP-J-mediated transcription. The intracellular domain of Notch4 transactivates RBP-J-mediated transcription, whereas the extracellular domain of Notch4 inhibits ligand-induced Notch1 activation^26^. The phenotypes of *Notch4* KO pituitary are similar to those of DAPT-treated mice, suggesting that these phenotypes of *Notch4* KO mice are mediated through the loss of RBP-J-mediated transactivation by Notch4 intracellular domain. *Notch4* cooperatively functions with *Notch1* for the regulation of the vascular development. In the absence of *Notch1*, *Notch4* deficiency exacerbates the vascular defects, suggesting redundant roles between *Notch1* and *Notch4* in the anterior cardinal vein, yolk sac vessels and retinal vasculature^25,33^. The phenotypes of *Notch*4-deficient adult pituitary posterior lobe vessels are similar to those observed in *Notch4*-deficient early postnatal retinal immature vasculature^26^. The redundancy between *Notch1* and *Notch4* in retinal vessel development also supports the hypothesis that the function of Notch4 in the adult pituitary development is dependent on RBP-J-mediated transcription. The only difference between the *Notch4* KO mice and DAPT-treated mice was in the length of the affected larger radius blood vessels, which might be caused by inhibitory effects of Notch4 extracellular domain^26^.

Loss of RBP-J in immature retinal endothelial cells increased the number of tip cells, but decreased proliferation, leading to the reduction in the number of endothelial cells^19^. In contrast, loss of RBP-J in mature retinal endothelial cells enhanced proliferation of endothelial cells of veins and surrounding capillaries, leading to enlarged veins^18^ but did not affect arterial fate. The vascular phenotype of *Notch4*-deficient or DAPT-treated adult pituitary appears to be different from that previously reported for Notch signaling deficiency. *Notch4* deficiency or DAPT treatment decreased the larger radius vessels instead of causing enlarged veins, reduced the vascular network and induced arteriovenous transformation in the adult pituitary. This suggests the phenotype is similar to that of immature retinal endothelial cells rather the mature retinal endothelial cells.

In adult retina, Dll4 and Notch1 have been reported to maintain blood-retinal barrier through arterial transcytosis suppression^34^. Retinal endothelial Notch signaling suppresses caveolar-mediated transcytosis through the reduced arterial PLVAP expression by blocking VEGFR2 signaling. In the adult retina, Notch modulates arterial barrier functions but not affects arteriovenous fates. In contrast, no alteration in PLVAP expression was detected in the adult pituitary gland of Notch4-deficient or DAPT-treated mice. These observations suggest the possibility of tissue-specific vascular regulation by Notch signaling.

## Conclusion

Our study suggests that Notch4 functions in the adult pituitary posterior lobe vasculature is dependent on Notch4 intracellular domain. It has been reported that the intracellular and extracellular domains of Notch4 exhibit opposing roles^26^. The phenotypic similarities between *Notch4* KO pituitary and DAPT-treated pituitary posterior lobes suggest that Notch4 intracellular domain, rather than its extracellular domain plays a key role in the adult pituitary vasculature.

Next, our findings suggest that the adult pituitary posterior lobe vasculature retains a responsiveness similar to that of immature blood vessels. Previous studies have reported the absence of Notch signaling reduced vascular growth in the developmental stage or in the tumor. In contrast, in mature adult vessels, Notch signaling is required for maintaining endothelial cell quiescence in veins and perivenous capillaries, and the loss of Notch signaling do not affect adult arterial fate. In contrast, the loss of Notch4 and DAPT treatment reduced vascular networks and affected vessels arteriovenous fate in the adult pituitary posterior lobes. These observations suggest distinct roles of Notch4 signaling of adult pituitary posterior lobe vascular maintenance. Notch signaling might affect the pituitary functions through the regulation of the homeostasis of adult vasculature network, similar to the way VEGF regulates the endocrine system through vascular maintenance^21^. The therapeutic potential of anti-Notch4 antibodies in tumor treatment has been demonstrated in murine model of breast cancer^35^. The systemic administration of anti-Notch4 antibodies may inadvertently compromise the integrity of these vessels, potentially leading to functional impairments. Therefore, careful evaluation of tissue-specific vascular responses and unintended effects on endocrine-associated vasculature is warranted to ensure the safety of Notch4-targeted therapies.

## Methods

### Animals

All animals used in this study were maintained and handled in accordance with the protocols approved by the Committee on Animal Research at Research Institute, Shiga Medical Center (Protocol No. R4-03). Mice were maintained at a controlled ambient temperature of 22 °C with 50% ± 10% relative humidity on a 12 h light/dark cycle, and was provided with food and water ad libitum. The animal experiments were carried in accordance with the ARRIVE (https://arriveguidelines.org) and relevant guidelines. The study was approved by the Committee on Animal Research at Research Institute, Shiga Medical Center (Protocol No. R4-03).

The Notch4tm1(KOMP)Vlcg mice line was kindly provided by Prof. Sally Dunwoodie^26^. Given that the concentration of DAPT reportedly decreases to one-fifth of its peak within 12 h post-administration^36^ two to three-month male C57BL/6 mice (*n* = 5/group) were injected with DAPT 100 mg/kg subcutaneously twice a day (9:00 AM and 9:00 PM) for 3 days. As a control, the same volume of vehicle (0.02% DMSO) was injected and sacrificed 12 h after the last injection. To harvest tissues, mice were euthanized by CO2 after anesthesia with inhaled isoflurane, with care to minimize pain. Pituitary glands collected from five different sets of mice were subjected to immunohistochemical staining.

### Immunohistochemical analysis

Dissected pituitary glands were fixed in 4%PFA for another 30 min and immersed in 30% sucrose for 12 h. The fixed pituitaries were embedded in Tissue-Tek O.C.T. Compound (Sakura Finetek) and cut at 30 μm thickness. Immunostaining was performed with the following antibodies: Alexa Fluor 568-conjugated isolectin GS-IB4 from Griffonia simplicifolia (1:100; I21412, Thermo Fisher Scientific, MA, USA), rabbit anti-Dll4 (1:200; ab7280, Abcam, Cambridge, UK), rat anti-plasmalemma vesicle associated protein (PLVAP; 1:100; ab27853, Abcam, Cambridge, UK), rat anti-EphB2 (1:100; MAB467, R&D systems, Minneapolis, USA), goat anti-EphB4(1:50;, AF446, R&D systems, Minneapolis, USA) and anti-Notch4 -APC (1:200; 128413, Biolegend, SanDiego CA, USA) and rabbit anti-NR2F2 (COUP-TF2) (1:250; ab211777, Abcam, Cambridge, UK). was used to label endothelial cells. Briefly, cryosections were incubated with primary antibodies for 24 h at 4 ℃, and then with secondary antibodies for 1 h at room temperature. Anti-species IgGs conjugated with Alexa 488 or Alexa 647 (A21206, A21247, Thermo Fisher Scientific, MA, USA) were used for secondary antibodies. Fluorescence images were obtained with a z-step of 1–2 μm using a SP8 confocal laser scanning microscopy (Leica) equipped with a 20 × objective lens (NA, 0.7) HC Plan APO 20X/0.70, Leica) and LAS X software (Leica). The image analysis was performed with Fiji software and analyzed on flattened Z-projections.

### Volumetric measurement of the pituitary posterior lobe

The fixed pituitaries were embedded in Tissue-Tek O.C.T. Compound (Sakura Finetek) and cut at 30 μm thickness. Images of serial sections through the entire pituitary posterior lobe were analyzed in Image-J. The interval between adjacent sections on each slide was 180 μm. Borders of the posterior lobe were traced manually followed by manual tracing.

### Vascular analysis

Sagittal sections of pituitary glands stained with isolectin B4 conjugated with Alexa 568 were analyzed with Fiji software. Isolectin B4-positive blood vessels of the posterior lobe were analyzed for vessel branching analysis, as previously described^29,30^. Briefly, the manually-delineated images of the pituitary posterior lobes were filtered with anisotropic diffusion 2D filter^37^ and processed with “tubeness” filter (sigma: 4), binarized with near peak values of thresholds to match blood vessels, and the vascular area was measured^38^. The “tubeness” filter is superior, particularly in the efficient extraction of dimmer vessels compared to other filtering methods. Binarized images were skeletonized and the number of vascular branches and branching points per 10,000 µm^2^ was counted by the Fiji plug-in “Analyze Skeleton(2D/3D)”. Next, binarized images were processed to Euclidean distance maps (EDM) of vessel voxel distance to the nearest background voxel, using the Fiji plug-in “Distance Map”. Vascular centerline pixels were extracted and the vessel radii were quantified by multiplying EDM with skeletonized images. The data were analyzed by Student’s t-test and considered to be significant when *P* < 0.05. Results are given as means ± S.E.M.

### Real-time RT-PCR

Total RNA was extracted from pituitaries using a RNeasy Mini Kit (QIAGEN, Valencia, CA, USA) and subsequently reverse-transcribed using a PrimeScript 1st strand cDNA synthesis kit (Takara, Shiga, Japan). Intron spanning *Taq*man probes were designed using the Roche Universal Probe Library method. Quantitative PCR reactions were run in a LightCycler 480 system (Roche-Diagnostics, Basel, Switzerland). All data were normalized to GAPDH expression levels measured with Universal ProbeLibrary Mouse GAPDH Gene Assay (Roche-Diagnostics, Basel, Switzerland).

The following primers were employed: *Notch1*: 5’-ctggaccccatggacatc-3’ and 5’- aggatgactgcacacattgc-3’, probe:#80; *Notch2*: 5’- tgcctgtttgacaactttgagt-3’ and 5’- gtggtctgcacagtatttgtcat-3’, probe:#6; *Notch3*: 5’- agctgggtcctgaggtgat-3’ and 5’- agacagagccggttgtcaat-3’, probe: #9; *Notch4*: 5’- ggacctgcttgcaaccttc-3’ and 5’- cctcacagagcctcccttc-3’, probe: #34; *Hes1*: 5’-tgccagctgatataatggagaa-3’ and 5’-ccatgataggctttgatgacttt-3’, probe: #20; *Hey1*: 5’-catgaagagagctcacccaga-3’ and 5’-cgccgaactcaagtttcc-3’, probe: #17; *HeyL*: 5’-ctgaattgcgacgattggt-3’ and 5’-gcaagacctcagctttctcc-3’, probe: #25.

### Statistical analysis

All data are expressed as means ± standard error of the mean. P values and all graphs were generated using Excel or StatView. Statistical significance was calculated using two-tailed unpaired Student’s t test. Significance was considered at *P* < 0.05. Repeated measures of ANOVA were employed to analyze vessel number distributions across branch length. Bonferroni’s post hoc tests were performed for pairwise comparisons. Normal distribution was assessed using Shapiro-Wilks normality test.

## Supplementary Information

Below is the link to the electronic supplementary material.

Supplementary Material 1

## Data Availability

The datasets used and/or analyzed during the current study available from the corresponding author on reasonable request.
