# Supplementary material for: Notch4 is essential for the maintenance of vascular homeostasis in the young adult pituitary posterior lobes
Source: Sci Rep. 2025 Aug 24;15:31153. doi: 10.1038/s41598-025-17225-5 (PMC12375735; doi:10.1038/s41598-025-17225-5)
Supplement: Supplementary file 1 — Supplementary Material 1 [file 41598_2025_17225_MOESM1_ESM.docx]

**Supplementary Fig.1**

**Supplementary Fig.1 The image processing method to quantitate vascular density, branch length, branching points, and branch radii.** To examine the vascular development of the pituitary posterior lobe, a region of interest (ROI) was manually delineated. After ROI extraction, vascular density was quantified. Euclidean distance maps were combined with vascular skeletons to quantify radius in skeletonized distance maps, which are also used to quantify vessel length, branching points (yellow *).
